# Survival in macaroni penguins and the relative importance of different drivers: individual traits, predation pressure and environmental variability

**DOI:** 10.1111/1365-2656.12229

**Published:** 2014-05-21

**Authors:** Catharine Horswill, Jason Matthiopoulos, Jonathan A Green, Michael P Meredith, Jaume Forcada, Helen Peat, Mark Preston, Phil N Trathan, Norman Ratcliffe

**Affiliations:** 1 British Antarctic SurveyHigh Cross, Cambridge, CB3 0ET, UK; 2 Institute of Biodiversity, Animal Health & Comparative Medicine, University of GlasgowGlasgow, G12 8QQ, UK; 3 School of Environmental Sciences, University of LiverpoolLiverpool, L69 3GP, UK

**Keywords:** bottom-up, demography, El Niño/Southern Oscillation, fledging mass, giant petrel, intrinsic factors, predation, sea surface temperature, southern annular mode, top-down

## Abstract

1. Understanding the demographic response of free-living animal populations to different drivers is the first step towards reliable prediction of population trends.
2. Penguins have exhibited dramatic declines in population size, and many studies have linked this to bottom-up processes altering the abundance of prey species. The effects of individual traits have been considered to a lesser extent, and top-down regulation through predation has been largely overlooked due to the difficulties in empirically measuring this at sea where it usually occurs.
3. For 10 years (2003–2012), macaroni penguins (*Eudyptes chrysolophus*) were marked with subcutaneous electronic transponder tags and re-encountered using an automated gateway system fitted at the entrance to the colony. We used multistate mark–recapture modelling to identify the different drivers influencing survival rates and a sensitivity analysis to assess their relative importance across different life stages.
4. Survival rates were low and variable during the fledging year (mean = 0·33), increasing to much higher levels from age 1 onwards (mean = 0·89). We show that survival of macaroni penguins is driven by a combination of individual quality, top-down predation pressure and bottom-up environmental forces. The relative importance of these covariates was age specific. During the fledging year, survival rates were most sensitive to top-down predation pressure, followed by individual fledging mass, and finally bottom-up environmental effects. In contrast, birds older than 1 year showed a similar response to bottom-up environmental effects and top-down predation pressure.
5. We infer from our results that macaroni penguins will most likely be negatively impacted by an increase in the local population size of giant petrels. Furthermore, this population is, at least in the short term, likely to be positively influenced by local warming. More broadly, our results highlight the importance of considering multiple causal effects across different life stages when examining the survival rates of seabirds.

## Introduction

Understanding the factors that explain changes in population size is central to population ecology, wildlife management and conservation biology. The trajectory of all living populations is determined by several demographic processes, and fluctuations in any one of these can affect the speed with which a population grows or declines. The regulatory mechanisms underpinning these processes are often complex and dynamic, and whilst many studies spanning many species have identified different drivers, few have assessed intrinsic factors, bottom-up and top-down mechanisms simultaneously to provide an insight into their relative importance (see Reid *et al.* 2013). Even fewer have investigated how these effects, when considered in unison, may vary between different life-history stages (see Schwarz *et al.* 2013).

There are many studies suggesting that oceanic predators experience bottom-up control of survival rates where the proposed mechanism is an effect of environmental variability on food availability. For seabirds, these relationships have been inferred for several species (Jenouvrier 2013). The methodological challenges of observing seabirds across large spatial scales and through unobservable life stages (e.g. deferred reproduction) have meant that top-down effects and early life stages have been largely precluded from these studies (Baum & Worm 2009). This is despite predation of seabirds being widely implicated as a potential driver of their population trends (e.g. Andersson 1976; Reisinger, De Bruyn & Bester 2011), and a growing body of literature highlighting that sub-adult life stages play a key role in shaping their population dynamics (Nur & Sydeman 1999).

As a well-studied seabird that has exhibited dramatic declines in population size (Borboroglu & Boersma 2013), the macaroni penguin is an ideal species for studying multiple regulation effects. Furthermore, they are one of the most important avian marine consumers in the sub-Antarctic region, reported to consume more prey than any other seabird species (Brooke 2004). Previous studies on this species have linked environmental covariates with the short-term population trajectory, where the proposed mechanism is an effect on reproductive performance (Reid & Croxall 2001; Forcada & Trathan 2009). However, little is currently known about how bottom-up regulation influences their survival rates, or what role individual traits and top-down regulation have to play. Here, we provide the first robust estimates of age-specific survival rates in macaroni penguins. Our analysis avoids the biases associated with flipper banding (Saraux *et al.* 2011a) and is the first seabird demography study to use mark–recapture modelling approaches to simultaneously consider and demonstrate the influence of individual traits, bottom-up and top-down regulation.

## Methods

### Demographic data

For 10 consecutive breeding seasons between 2003 and 2012 (2003 refers to the breeding season spanning November 2002 to April 2003, and so on), adult and fledgling macaroni penguins at Bird Island, South Georgia, were implanted with sterile 32-mm passive integrated transponder (PIT) tags (Texas Instruments, USA; www.ti.com/lit/ds/symlink/ri-trp-ir2b.pdf). PIT tags were implanted under the skin half way down the back (see TableS1, Supporting information for annual sample sizes in relation to population size and fecundity). Within each year, all chicks were PIT-tagged on a single day (16–19th February) shortly before synchronous fledging during mid- to late February. Chicks were selected from the periphery of the crèche; at this stage in the breeding season chicks abandon their individual nest sites during the day in order to ‘crèche’ together. As the crèche is highly dynamic (especially in response to disturbance), and the sample of chicks tagged was proportionally large in comparison to the number raised to fledging, the sample was considered to be representative of the whole colony (TableS1). In order to minimize disturbance, adults were PIT-tagged periodically throughout the breeding season by capturing them as they entered the colony.

As macaroni penguins are entirely pelagic during the winter months and forage away from the continental shelf zone of South Georgia (Ratcliffe *et al.* 2014), survival rates were inferred by ‘recapturing’ individuals during the breeding period. Birds were recaptured using an automated gateway PIT reader system, as described by Green, Trathan & Preston (2006). The gateway houses two antennae that electromagnetically activate the PIT tags, scan the unique identifier and store the data to memory. The gateway is situated at the single entrance to the colony, and independent tests have shown reliable recording of individual presence when operational (Green, Trathan & Preston 2006). Data from manual recapture using hand-held PIT scanners were also included for all seasons; however, these data were a by-product of studies with aims other than estimating survival rates and therefore were not conducted across the entire colony. Gateway operation was variable between years, and during 2007, the gateway failed completely so that all recaptures were made manually (Table S1).

### Covariates

To enable a direct comparison of covariate effect size from their regression coefficients, each variable was standardized to 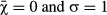 (Schielzeth 2010).

#### Individual traits

To examine the within-year effect of individual traits on survival, we used individual body mass at PIT tagging as a proxy for mass at fledging. All birds were weighed to the nearest 0·05 kg using a Pesola spring balance. These data were included in the capture histories for birds tagged as chicks. The between-year effect of fledging mass was examined using the annual mean as a time-dependent covariate. The year-to-year variation in observed fledging mass was examined using an analysis of variance (anova) model performed in the program R (v. 2.15.3).

#### Top-down

Northern *Macronectes halli* and southern *M. giganteus* giant petrels are large birds that are predators and scavengers. At several locations penguins form a major component of their breeding season diet (Bonner & Hunter 1982; Hunter & Brooke 1992). At Bird Island, this is thought to consist predominantly of adult macaroni penguins (Hunter 1983). More recently, anecdotal accounts from Bird Island report macaroni penguin chicks being heavily predated by giant petrels as they fledge (J. A. Green; P. N. Trathan, pers. obs.; R. A. Phillips, pers. comm.). Reports detail chicks being drowned at sea shortly after they enter the water, as well as being attacked on land whilst transiting from the colony to the shore. This behaviour is consistent with reports for other penguin species, where predation by giant petrels is reported to take place on land and from the water's surface when leaving or approaching the beach (Le Bohec *et al.* 2003; Ryan, Sommer & Breytenbach 2008).

On Bird Island, giant petrels breed sympatrically at densities that at the time of publication were among the highest in the world. Nesting pairs in three study areas were visited weekly during the incubation and chick-rearing stages to determine individual breeding success. The number of giant petrel chicks successfully reared to fledging was used as a proxy for giant petrel predation pressure. This measure relates to the number of giant petrels that would still be under central place constraint and therefore foraging locally when the penguin chicks fledge.

#### Bottom-up

**Fig 1:**
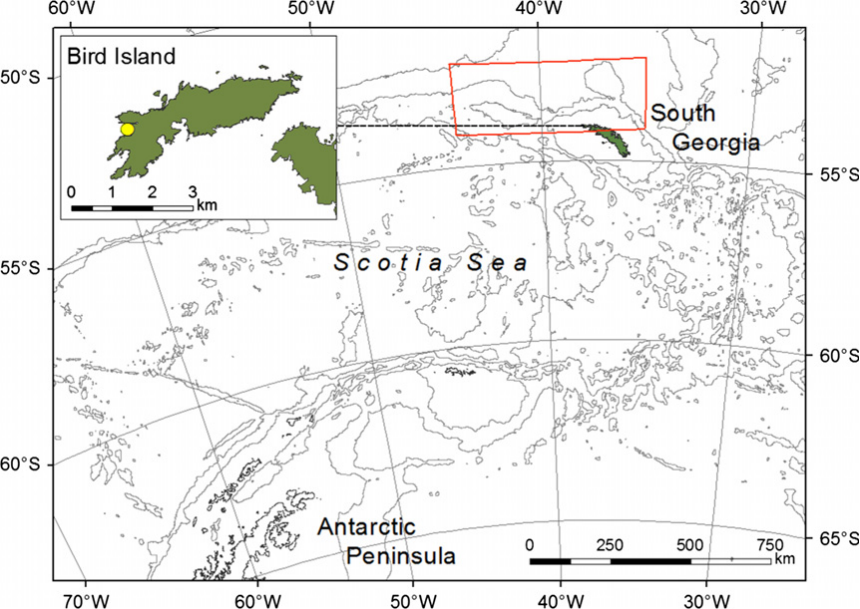
Map of Bird Island in relation to the Antarctic Peninsula. Bird Island with study colony shown within the insert map, dashed line to location at South Georgia. Bathymetry contours at 1000, 3000, 5000 m. Spatial scale of LSST covariate shown by red square.

We quantified the effect of one local and two quasi-remote environmental variables: (i) local sea surface temperature anomalies in the region associated with foraging during the breeding season (Fig.1; 35·5°W to 44·5°W; 52·5°S to 54·5°S) (LSST); (ii) the El Niño/Southern Oscillation (ENSO) phenomenon; and (iii) the Southern Annular Mode (SAM).

LSST reflects local environmental conditions and is used here as a proxy for diverse processes that are thought to influence prey retention and survival within the continental shelf zone; such as oceanic advection and upwelling (Murphy *et al.* 1998; Trathan *et al.* 2003). Antarctic krill *Euphausia superba* (an important component of macaroni penguin diet at South Georgia; Waluda *et al.* 2012) are not thought to be self-sustaining in this region, but are exported from spawning grounds and transported to South Georgia via advection (Murphy *et al.* 1998). The Antarctic Peninsula, the South Orkney Islands and the Weddell Sea have all been highlighted as potential source regions (Siegel 2005), although modelling studies also show that oceanic waters right across the Scotia Sea offer suitable conditions for krill spawning and larval development (Hofmann & Hüsrevoğlu 2003). The large-scale distribution of krill around South Georgia is thus a function of production (recruitment and growth) and dispersal from spawning grounds, as well as retention and mortality in the study region (Murphy *et al.* 2007). We used LSST data calculated according to methods described by Reynolds *et al*. (2002) and obtained from the National Oceanographic and Atmospheric Administration (NOAA) through the International Research Institute. The data are optimum-interpolation LSST values, produced monthly on a one-degree grid using *in situ* and satellite LSST, plus LSST estimated from sea-ice cover. Data are available at http://iridl.ldeo.columbia.edu/SOURCES/.NOAA/.NCEP/.EMC/.CMB/.GLOBAL/.Reyn_SmithOIv2/.monthly/.SSTa/.

The remote environmental variables (ENSO and SAM) offer proxies for remote forcing on LSST, in addition to other environmental variables throughout the Southern Ocean (Meredith *et al.* 2005, 2008). ENSO is a major mode of coupled atmosphere ocean variability that operates on interannual timescales. Whilst it is triggered in the equatorial/tropical Pacific, it has teleconnections to the Southern Ocean and Antarctica via both atmospheric and oceanic processes (Turner 2004). SAM is the dominant mode of extra-tropical variability in the Southern Hemisphere and is characterized by shifts in atmospheric mass between a node centred over Antarctica and a ring encompassing the lower latitudes. SAM fluctuates on timescales of weeks to decades, including interannual periods, and is associated with variations in the circumpolar winds over the Southern Ocean (Sengupta & England 2006). As physical variability in this region is linked with the extent of sea ice during the winter, we used a summer mean of the remote climatic covariates, which corresponds to the breeding season when birds are under strong central place constraints. Whilst teleconnections between LSST, ENSO and SAM at South Georgia mean that these variables are not independent, the relationship is highly dynamic over time, precluding the use of principal component analysis. For ENSO, we used the bivariate ENSO time series (BEST) index (Smith & Sardeshmukh 2000). This is broadly equivalent to the Southern Oscillation Index (SOI), which has been used previously as a proxy for ENSO variability (e.g. Jenouvrier, Barbraud & Weimerskirch 2005). The BEST index offers advantages as it also includes an oceanic component (Niño 3.4 LSST index) as opposed to being derived from two atmospheric stations only (Smith & Sardeshmukh 2000). Monthly values of the BEST index were obtained from the Climate Diagnostics Center of NOAA (data available at http://www.cdc.noaa.gov/people/cathy.smith/best/), and monthly values of the SAM index were obtained from the Climate Prediction Center of NOAA (obtained from http://www.cpc.ncep.noaa.gov/).

Candidate temporal lags were calculated from published information by summing plausible physical and biological process lags (Table S2, Supporting information). Whilst a positive phase of the SAM is associated almost immediately with warm LSST anomalies in the study region (at time lags of ∼1 month), positive ENSO values generally take 1·5–2 years to appear as warm LSST anomalies in the Scotia Sea (Meredith *et al.* 2008). Biological lags associated with the recruitment of krill to South Georgia were added to the physical lags in two potential spawning and dispersal scenarios. Either spawning occurs across the Scotia Sea with recruitment maintained within that year in all shelf regions (Brierley *et al.* 1999), or spawning and successful survival through the first year occurs mainly in central and southern areas of the Scotia Sea with dispersal occurring through interactions with the ocean and sea ice over the next 1–2 years (Hofmann *et al.* 1998).

### Data analysis

#### Capture–mark–recapture modelling of survival

Construction of individual capture histories was carried out using R and Microsoft Access (Cooch & White 2011). The interval between sampling intervals was 1 year, from the end of the moulting period in year_*t*_ to the end of the moulting period in year_*t*+*1*_ (1st May of year_*t*_ to 30th April of year_*t+1*_; macaroni penguins renew their feathers at the end of the breeding season). As chicks fledge the colony before the moulting period the survival interval for this age class started slightly earlier; however, survival rates are considered to represent the fledging year interval, also finishing in the subsequent year at the end of moult. Separating survival rates between the breeding and interbreeding season was not considered as part of this study.

The data set contained 966 individuals PIT tagged at age 1 or above and 1070 individuals PIT tagged at fledging (Table S1, Supporting information). Any individuals PIT tagged at fledging without mass data were removed from the analysis (*n* = 11; 7 birds from 2009, 4 birds from 2011). Annual survival rates were estimated using a multistate capture–mark–recapture model fitted using Program mark (Lebreton & Pradel 2002). In order to account for the heterogeneity in recapture rates associated with deferred reproduction, individuals within the population were classified into two states. State 1 included all birds PIT tagged at fledging that have not yet returned to the colony (the ‘unobservable state’). State 2 included birds tagged at age 1 or above and birds tagged at fledging that have since returned to the colony and are assumed to be available for recapture on an annual basis (the ‘observable state’). This approach simplifies the model structure by allowing individuals to return to the colony for the first time at different ages, as opposed to using complex interactions to describe age-dependent recapture rates (e.g. Spendelow *et al.* 2002).

The starting model (here on referred to as the ‘global model’) was constructed to have the largest number of parameters that was biologically meaningful to the study system whilst also providing adequate fit to the data. The goodness-of-fit of this model was assessed using the overdispersion parameter, median *ĉ*, as this offers the most suitable approach for multistate models with age-specific transitions (Cooch & White 2011). Median *ĉ* was estimated with a lower bound of 1·0, an upper bound of 4·0 and 100 replicates, and a score greater than 3 was taken to represent lack of fit (Lebreton *et al.* 1992). The error of all candidate models was increased by this value during model selection. The global model structure incorporated age-specific variation in transition rates from the unobservable to the observable state; state and time-specific variation in recapture rates; and an interaction between age and time-specific variation in survival rates. All candidate models were nested within this model, and the most parsimonious was selected using the second-order Akaike information criterion (AIC_c_) (Burnham & Anderson 2002). A difference of less than 2 AIC_c_ units was taken to suggest that competing models received a similar amount of support from the data. In this case, the model with fewer parameters was selected through parsimony. A difference of more than 2 indicated strong support for the model with the lower AIC_c_ (Burnham & Anderson 2002).

#### Transition probability: age of first return to the colony (Ψ)

Age of first return to the colony was modelled with a maximum at 3, 4 and 5 years old. The model thus estimated the probability that if an individual survives until year *t* and age *i*, they will return to the colony for the first time at age *i*, up to the respective maximum age. The probability of birds older than these maxima returning for the first time was fixed to a value of one (Spendelow *et al.* 2002). Since birds entering the observable state are then assumed to be available for recapture on an annual basis, the reverse transition back into a deferred reproduction state was fixed to a value of zero (Spendelow *et al.* 2002). Model simplification involved fitting age of transition as a linear and natural logarithm trend. This allowed the relationship between age and transition probability to change according to a function, reducing the number of parameters necessary to model this rate from 4 to 2. Incomplete attendance histories at the individual level, caused by variable operation of the gateway PIT reader within seasons, precluded the identification of individual recruitment to the breeding population from seasonal attendance patterns (see Le Bohec *et al.* 2007).

#### Recapture probability (*p*)

The recapture probability of birds in the unobservable state was fixed to zero (*p0*). For birds in the observable state, recapture probability was allowed to vary from year to year (*p*_*t*_). Model simplification involved fitting a reduced time-dependent variable with seasons grouped manually based on the duration the gateway PIT reader was in operation (*p*_T3_): (i) not working (0 days: 2007); (ii) working intermittently (1–100 days: 2009); and (iii) working well (>100 days: all other years) (Table S1). A constant recapture probability through time was also tested.

#### Survival probability (ϕ)

Survival was modelled with three separate age classes (a3: fledging year, second year and all years thereafter), two separate age classes (a2: fledging year and all years thereafter) and without age effects (a1). The effect of age and time was tested as additive and interactive with age class. After selecting the best age structure, the yearly variation in survival probabilities was modelled as an age-specific function of individual- and year-specific covariates using a step-up approach. The influence of individual traits (fledging mass; for birds marked as chicks only) was tested in isolation using a likelihood ratio test (LRT; Skalski, Hoffmann & Smith 1993). Each year-specific covariate was then fitted alongside individual fledging mass and the most significant retained. This process was iterated until the addition of any remaining year-specific covariates did not significantly improve the amount of deviance explained by the model. The proportion of deviance accounted for by the inclusion of each additional covariate (*R*^2^_DEV) was calculated as [DEV(Model.) − DEV(Model_cov_)/DEV(Model.) − DEV(Model_*t*_)], where DEV was the deviance for models with constant (.), covariate (cov) and total temporal (*t*) variation (Skalski, Hoffmann & Smith, 1993). The model including the covariate selected during the previous step was used as the constant model. A covariate explaining more than 20% of the remaining deviance was taken to be significantly influential (Grosbois *et al.* 2008). The statistical support for a covariate effect was also measured using ANODEV in Program mark (Skalski, Hoffmann & Smith, 1993). Finally, the significance of each covariate retained by the best candidate model was verified through the confidence interval of its logit-scale regression coefficient. Variance component analysis was used to assess the total time-dependent process variation (σ) in the survival rates of each age class (Cooch & White 2011).

### Sensitivity analysis

To assess the relative importance of each covariate within the best candidate model, we compared covariate effect sizes and calculated the partial derivative of survival with respect to each variable in a sensitivity analysis. For example, in a model where fledgling mass (Mass) is the only explanatory variable retained, the sensitivity (*S*) of survival (ϕ) to mass would be calculated as:
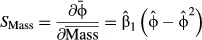
where 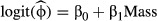.

## Results

### The global model

For the global model with three age classes and maximum age of transition at 5 years old, median *ĉ* was estimated at 2·96 (SE = 0·02) (Table1: model 8) (see Table1 and Table S3, Supporting information for all other candidate models). The addition of further age classes within the global model structure led to a substantial loss of fit (median *ĉ *>* *3).

**Table 1:** Modelling capture, survival and transition probabilities for macaroni penguins (2003–2012)

No.	Model	AIC_c_	ΔAIC_c_	*k*	Model deviance
1	ϕ_*a2 a+t/a+t*_ *p*_*0/T3*_ Ψ_4 ln/0_	1816·37	0·00	18	1786·27
2	ϕ_a2 a^*^t/a^*^t_ *p*_*0/T3*_ Ψ_4 ln/0_	1817·77	1·40	26	1769·53
3	ϕ_a2 a^*^t/a^*^t_ *p*_*0/t*_ Ψ_4 ln/0_	1817·81	1·44	31	1767·55
4	ϕ_a2 a^*^t/a^*^t_ *p*_*0/t*_ Ψ_4 linear/0_	1820·72	4·35	31	1770·46
5	ϕ_a2 a^*^t/a^*^t_ *p*_*0/t*_ Ψ_5/0_	1821·28	4·91	34	1762·94
6	ϕ_a2 a^*^t/a^*^t_ *p*_*0/t*_ Ψ_4/0_	1823·01	6·64	33	1766·69
7	ϕ_a2 a^*^t/a^*^t_ *p*_*0/t*_ Ψ_3/0_	1843·22	26·85	32	1790·95
8	ϕ_a3 a^*^t/a^*^t_ *p*_*0/t*_ Ψ_5/0_	1872·68	56·31	42	1814·34
9	ϕ_*a1 t*_ *p*_*0/t*_ *Ψ*_5/0_	1931·00	114·63	25	1890·83
10	ϕ_a2 a^*^t/a^*^t_ *p*_*0/*._ Ψ_4 ln/0_	2411·78	595·41	23	2373·63

Model fit is assessed using the lowest AIC_C_ with the difference between the best candidate model and other models specified (ΔAIC_c_). See Table S3 for global models considered for age classes and maximum age of transition. Here, *k* is the number of parameters in the statistical model. Explanation of model notation (Cooch & White 2011): (ϕ) refers to the structure used to model survival probabilities, (*p*) refers to capture probabilities, and (*Ψ*) refers to transition probabilities. *p* and *Ψ* probabilities are separated by a forward slash to show the model structure for each state, unobservable/observable; ϕ probabilities are separated by a forward slash to show the model structure for each age class, fledgling year/older than 1 year. (a) shows whether the function refers to a 1, 2 or 3 age class structure. (*t*) indicates a fully time-dependent structure; (a^*^t or *a *+ *t*) a fully time-dependent structure that is interactive or additive with age; (·) a constant structure; and (0) a fixed probability at zero.

### Age of first return to the colony

**Fig 2:**
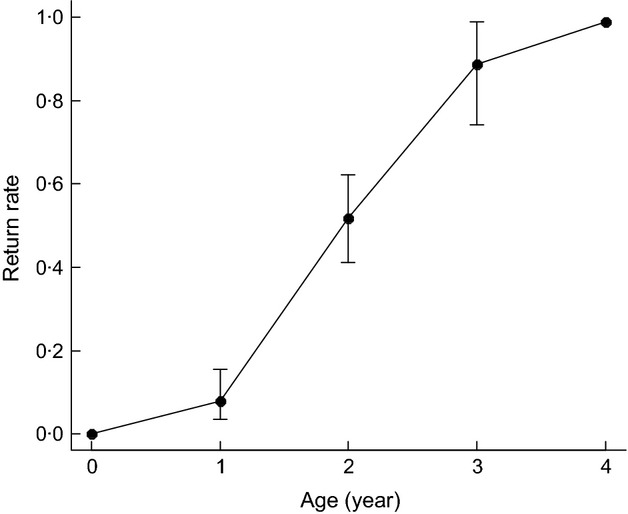
Cumulative rate of first return to the colony at different ages (as a natural logarithm function with 95% confidence intervals).

Virtually all birds had transited to the observable state by age 4 years (Table1: model 6 vs. 5,7, Fig.2, Table S3). The overall trend for rate of first return was best described by a natural logarithm function (Table1: model 3 vs. 4,6, Fig.2). The rate of birds returning to the colony by age 1 was low (0·1; 10% of the population), increasing dramatically so that cumulatively 89% had returned by age 3 (Fig.2).

### Factors affecting recapture rates

There was high variability in the annual recapture probability of observable birds (Table1: model 3 vs. 10). This was best explained by the annual variation in the number of days that the gateway PIT reader was operational (Table1: model 2 vs. 3). Recapture rates were predominantly high when the gateway was in operation for more than 100 days (0·99); slightly reduced during years with intermittent operation (0·88); and considerably lower when reliant on manual recaptures only (0·15).

### Factors affecting survival rates

**Fig 3:**
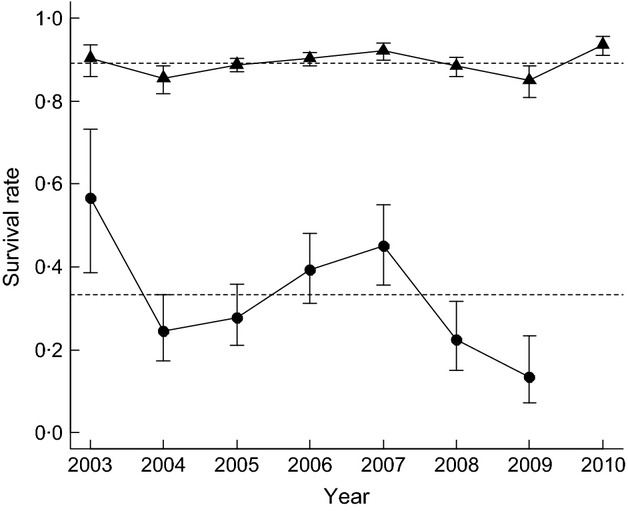
Survival rates of macaroni penguins; fledging year (black circles) and birds older than 1 year (black triangles). Age-specific means are shown with dashed lines.

The annual variation in survival rates was best described in two age classes (Table1: model 5 vs. 8–9, Table S3), and the difference between these was additive with time (Table1: model 1 vs. 2). Survival rates were considerably lower and more variable during the fledging year (Fig.3; mean = 0·33, SE = 0·06, σ = 0·12) than in the older age class (Fig.3; mean = 0·89, SE = 0·01, σ = 0·01). Due to large confidence intervals associated with the proportion of birds still in the unobservable state, the fledgling age class for 2010 is not presented.

**Fig 4:**
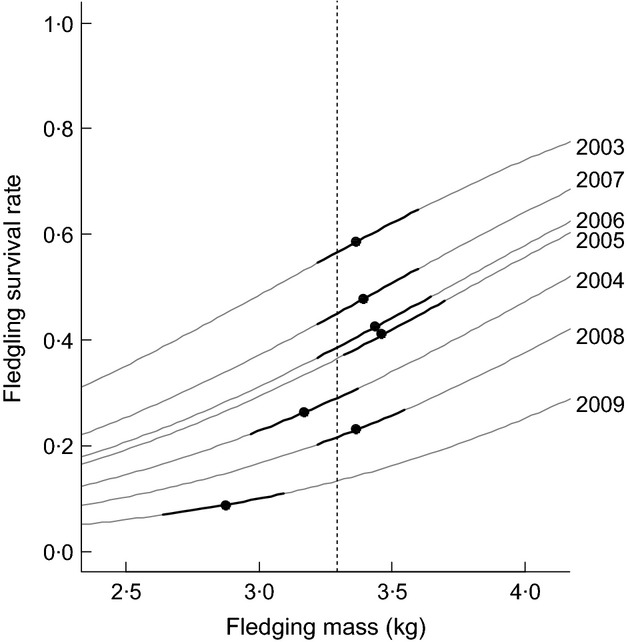
Fledgling survival rate modelled as a function of fledging body mass with the annual interquartile range (black line), annual mean (black circles) and overall mean (dashed line) shown for observed masses. Extrapolation of fledgling survival rate over a range of de-standardized values for fledgling mass (gray line) was carried out within Program mark.

The step-up procedure produced three significant variables influencing survival rates during the fledging year and two significant variables influencing the survival rates of birds older than 1 year; the results of this procedure are summarized in Table 2 (see Table S4, Supporting information for all other candidate models). Within a year, heavier chicks had consistently higher rates of survival than lighter ones so that the slope of this relationship is the same across years (Table 2, Fig. 4). However, the intercept term of this relationship was not adequately described by the interannual variation observed in fledging masses (Fig. 4: overall mean = 3·28; anova: *F*_7,814_ = 38·73, *P* < 0·001). For example, during 2009 considerably lower fledging masses and survival rates were documented, but during 2005 and 2008 low-to-average survival rates were documented despite fledging masses being average to high (Table 2, Table S4, Fig. 4).

**Table 2:** Step-up model selection procedure, ANODEV and LRT tests for individual trait, top-down and bottom-up covariates. At each step, only the most significant variable is shown. Recapture and transition probabilities specified as Model 1 (Table1)

Step	Model	*k*	d.f.	ANODEV tests for cohort-level covariates			LRT tests for individual-level covariates	
				*F*	*P*	*R*^2^	χ^2^	*P*
1	./.	10	–	–	–	–	–	–
2	Mass/.	11	1	–	–	–	11·00	<0·01
3	Mass + Pred./Pred. *	12	1	11·29	<0·01	0·43	–	–
4	Mass + Pred./Pred.	13	1	3·46	0·08	0·20	–	–
5	Mass + Pred. + LSST_−1_/Pred. + LSST_−1_	14	1	7·25	0·02	0·36	–	–
6	Mass + Pred. + LSST_−1_ + SAM_0_/Pred.+ LSST_−1_ + SAM_0_	15	1	1·46	0·22	0·12		
		–			n.s. » Stop			

Survival probabilities are separated by a forward slash to show the model structure for each age class, fledgling year/older than 1 year.

Unless specified predation pressure was included as an interactive effect with age class and environmental covariates as an additive effect with age class (see Table S4 for all other candidate models considered). (^*^) Predation pressure included as an additive effect with age class. (d.f.) degrees of freedom given as the difference in the number of estimable parameters between the two models compared using the step-up procedure. *R*^2^ (*R*^2^_DEV) gives the further deviance explained by adding a covariate.

**Fig 5:**
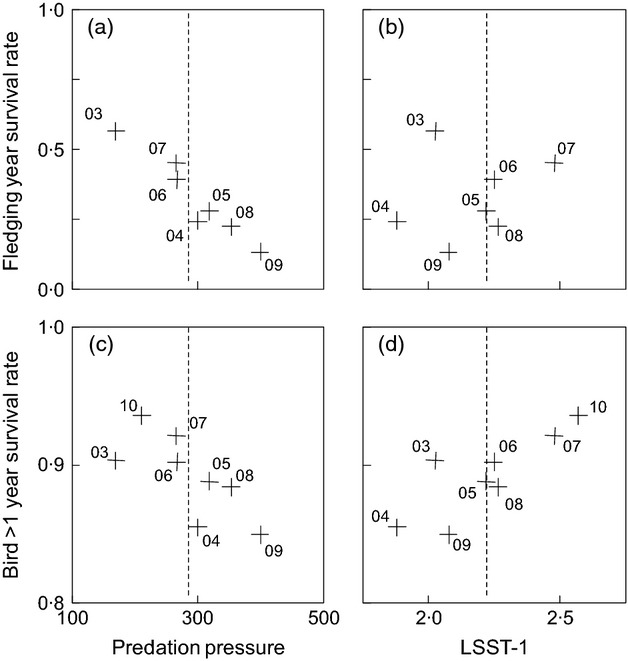
Survival of fledgling macaroni penguins in relation to a) predation pressure (mean shown with dashed line) and b) LSST_−1_ (mean shown with dashed line); Survival of macaroni penguins older than 1 year in relation to c) predation pressure and d) LSST_−1_. Cohorts labelled by season, and covariates incorporated as de-standardized. LSST included under specified lag.

The step-up procedure showed the year-to-year variation in survival rates of both age classes to be best described as a function of predation pressure and LSST_−1_ (Table 2). Although the interaction between predation pressure and age class was at the boundary of influence, this parameter was retained as anecdotal evidence reports a considerably higher incidence of giant petrel predation on fledgling chicks compared with adults (J. A. Green; P. N. Trathan, pers. obs.). Both age classes showed a negative response to predation pressure that was smaller in the older age class (Table 3, Fig. 5). Bottom-up regulation from LSST_−1_ was best incorporated as an additive effect that was positive for both age classes (Table 3). There was no evidence for any additional effects from LSST_0_, ENSO_−2_, ENSO_−3_, SAM_0_ or SAM_−1_ (Table S4: *R*^2^_DEV<0·2, *P* > 0·05).

**Table 3:** The relative importance of each covariate by age class, estimated using the partial derivative of survival with respect to each covariate

Age Class	Covariates		
	Fledging mass	Predation pressure	LSST_−1_
Fledgling	0·09	−0·15	0·03
>1 year old	–	−0·02	0·01

Survival rates during the fledging year were most sensitive to top-down predation pressure, followed by individual fledging mass, and finally bottom-up environmental effects (Table 3). In contrast, the older age class showed a similar response to fluctuations in bottom-up regulation and top-down predation pressure (Table 3). Consistent with the lower variance, the strength of these effects was much smaller on the older age class (Table 3).

## Discussion

### Age of first return to the colony and recapture rates

Despite operation of the automated gateway PIT reader being variable between years, this study obtained high detection rates when the gateway was functioning. This system is therefore highly suitable for examining otherwise unobservable life stages. The brief colony attendance of immature penguins means that conventional visual observation methods are likely to overestimate the age of first return. We found that more than 75% of immature birds had returned to the colony for the first time by age 3 and that 10% visited the colony in their first year. Based on surveys of banded birds, first breeding attempts of macaroni penguins are not thought to occur until age 6–8 (Williams 1995). However, other studies based on banded crested penguins have shown that first return to the breeding site coincides with age of first reproduction attempt (±1 year; Guinard, Weimerskirch & Jouventin 1998). As macaroni penguins may reach physiological maturity several years in advance of first breeding attempts (Williams 1992), further work is needed to define the mean age of recruitment for this population. An alternative explanation for birds returning earlier in life is to start prospecting future breeding sites (Boulinier *et al.* 1996).

### Factors affecting survival

#### Age

The survival rates of macaroni penguins were best described in a two age class structure. During the study period, birds typically experienced low and variable survival rates during the first year following fledging, and higher, more stable survival rates from age 1 onwards. Whilst it is not uncommon for seabirds to experience lower rates of survival during the fledging year (e.g. Frederiksen *et al.* 2008), other PIT-tagged penguins that breed in sub-Antarctica have shown fledgling survival rates to be more similar to birds older than 1 year (Saraux *et al.* 2011b). The rates reported here are more similar to species that predominately breed in temperate zones, such as those reported for PIT-tagged little penguins *Eudyptula minor* (0·05–0·60: Sidhu *et al.* 2012) and banded northern rockhopper penguins *Eudyptes moseleyi* (0·27: Guinard, Weimerskirch & Jouventin 1998). Given the large variation between our seven study cohorts of fledgling birds (2003–2009), with rates almost comparable with the older cohort during 2003, it is possible that given certain extrinsic conditions (i.e. a low predation pressure and an elevated LSST_−1_), fledgling macaroni penguins are capable of much higher levels of survival. The rates we report for the older age class correspond with those reported for other penguin species that breed in the sub-Antarctic region and are marked with PIT tags (0·82–0·96; Le Bohec *et al.* 2008; Dehnhard *et al.* 2013).

#### Individual traits

Within a given year, macaroni penguins fledging with heavier body masses consistently showed higher survival rates than birds fledging lighter. This agrees with previous work on penguin species marked with flipper bands (Olsson 1997; McClung *et al.* 2004). As flipper banding impairs survival rates (Saraux *et al.* 2011a), the consistency of this correlation in our study population (that are marked with PIT tags) further affirms that within a given year fledging penguins are highly dependent on their somatic resource whilst they gain the necessary foraging experience to search for patchily distributed prey (see also Saraux *et al.* 2011b).

Fledging mass of macaroni penguins is a function of growth rates driven by parent bird foraging and provisioning strategies (Barlow & Croxall 2002). Both fledging mass and survival rates were lower than average during 2004 and 2009, which coincides with years when macaroni penguin diet switched from being predominantly krill, to predominantly fish or *Themisto gaudichaudii* (Waluda *et al.* 2012). In addition, during 2004 parent birds from our study site were foraging further afield; an indication that they were targeting a different prey source (Trathan *et al.* 2006). A change in food supply is thus the most obvious explanation for the low masses observed during 2004 and 2009. However, the lack of a year-to-year relationship between fledging mass and survival indicates that this variable cannot be the only driver affecting the survival rates of this age class. Furthermore, as the individual fledging mass covariate remains significant in a model that also includes a bottom-up covariate, where the proposed mechanism incorporates food supply, we suggest that intrinsic traits such as structural size and physiology are also important when assessing individual quality.

#### Top-down effects

This is one of the first studies to illustrate how penguin survival rates may change under different levels of predation pressure from giant petrels. Predation pressure was shown to have a significant negative effect on both fledgling and adult macaroni penguins, albeit to a much lesser extent in adults. Lacking in experience, size and strength, fledgling birds are more susceptible to predation, and of the covariates considered, predation pressure had the strongest impact on survival during the fledging year. This was particularly apparent during 2005 and 2008, when average-to-high fledging masses and average environmental conditions coincided with elevated levels of predation pressure to produce lower-than-average survival rates.

Giant petrels are known to kill prey of similar and greater mass than an adult macaroni penguin (Le Bohec *et al.* 2003), and our results support anecdotal observations of direct predation (J. A. Green; P. N. Trathan, pers. obs.; R. A. Phillips, pers. comm.). Furthermore, this study corresponds with dietary analysis that shows macaroni penguins form a major component of giant petrel diet during the breeding season (Hunter 1983). Further work is needed to understand how giant petrels utilize different on-land prey resources (i.e. fur seal carcasses, penguins; Bonner & Hunter 1982; Hunter & Brooke 1992; González-Solís, Croxall & Wood 2000). Whilst giant petrels show species- and gender-driven segregation of foraging areas (González-Solís, Croxall & Wood 2000), it is unknown how this translates onshore during times of elevated resource, such as the synchronized fledging of penguin chicks. Under the observed conditions, we can predict that macaroni penguins are likely to be negatively affected by an increase in the population size of giant petrels, although the possibility of interference competition should be considered when making long-term projections. Finally, our study focuses on near-shore predation effects associated with the local giant petrel population. However, offshore predation by these and other species cannot be discounted despite being harder to empirically observe. There are near-shore observations of killer whales *Orcinus orca* (Pitman & Durban 2010), leopard seals *Hydrurga leptonyx* (Ainley *et al.* 2005) and otariid species (Bonner & Hunter 1982; Lalas *et al.* 2007) predating on various species of penguin, yet little is known about how these predators may influence survival rates during both the breeding and interbreeding period.

#### Bottom-up effects

Climatic change can have contrasting effects on different penguin populations and species (Croxall, Trathan & Murphy 2002). We infer from our results that macaroni penguins survival at South Georgia has a positive association with local warming. Whilst the majority of penguin climate demography studies report a negative association between climate warming and the survival rates of adult penguins (Barbraud & Weimerskirch 2001; Le Bohec *et al.* 2008), a positive effect has also been noted in specific populations of temperate and sub-Antarctic fledgling birds (Saraux *et al.* 2011b; Sidhu *et al.* 2012). It should also be kept in mind that LSST_−1_ does not necessarily drive survival rates to change; rather LSST_−1_ represents local environmental conditions and physical variability. Thus, each species could have optimum extrinsic conditions such that relationships between survival and environmental effects are nonlinear and possibly non-monotonic. The observed trend with warming will thus depend on whether the conditions at the study location are above or below the optimum (see Croxall, Trathan & Murphy 2002; Jenouvrier *et al.* 2012). Due to the positive relationship shown here, we suggest that the range of LSST values observed during the study period represents below optimum conditions for macaroni penguin survival. Determination of the point at which environmental conditions move across the optimum threshold would require a longer time series with greater variation in LSST.

It is also possible that LSST_−1_ is acting as a proxy for a separate process. This could be a remote effect associated with krill dispersal from the spawning ground, or a more local effect at South Georgia associated with krill retention and survival throughout the year. Atkinson *et al*. (2004) show a correlation between decreased levels of krill in the study area and reduced winter sea-ice extent further south. Here the proposed mechanism is a depletion of winter resources at the spawning grounds. In contrast, Loeb *et al*. (1997) show years of extensive sea ice increasing the retention of krill at the spawning ground, which may decrease the biomass of krill further upstream in the study area. Enhanced krill recruitment to South Georgia is therefore most likely to be associated with winters of average ice conditions (Quetin & Ross 2003). In terms of a local effect, conditions at South Georgia may control the proportion of krill that survive and are retained throughout the year. Despite krill density in these waters being lower during the winter months, concentrations are still substantial enough to support a seasonal fishery within the continental shelf zone (Trathan *et al.* 1998). Year-round local conditions are therefore likely to influence the biomass of the basal prey stock from one breeding season to the next (Murphy *et al.* 1998). Further work is needed to elucidate how local environmental conditions affect the resident prey species at South Georgia.

During the breeding season, the main foraging grounds of macaroni penguins from Bird Island overlap with the foraging grounds of Antarctic fur seals. The rapid recovery of fur seals following the cessation of their commercial exploitation (Trathan, Ratcliffe & Masden 2012) means they are now considered to be a major competitor for krill during the breeding season (Reid *et al.* 1996). Whilst we do not examine the effects of interspecific competition in this study, growth of the fur seal population at Bird Island stabilized and started declining prior to the study period examined here (1985–2000; Reid & Croxall 2001). Furthermore, as the winter foraging areas of these species are largely different (Staniland *et al.* 2012; Ratcliffe *et al.* 2014), increased levels of interspecific competition during the breeding season are more likely to affect population fecundity as opposed to survival rates.

## Conclusions

This study represents the first seabird demography study to use mark–recapture modelling approaches to simultaneously consider and demonstrate the influence of individual traits, top-down and bottom-up regulation. We show that interannual variation in survival rates of macaroni penguins at South Georgia is forced by a combination of individual quality, predation pressure and environmental variability. Under the observed conditions, we can predict that, at least in the short term, macaroni penguins will most likely be negatively affected by an increase in the population size of giant petrels and positively affected by local warming. Despite marked uncertainty in the long-term behaviour of the environmental variables considered, most IPCC class models predict continued warming in the Southern Ocean over the coming decades. We show that fledging macaroni penguins are most sensitive to fluctuations in top-down predation pressure, followed by the effects of individual traits and then bottom-up regulation. In contrast, birds older than 1 year have a similar response to fluctuations in bottom-up regulation and top-down predation pressure. The age-specific response of macaroni penguins to the physical and biological processes considered here confirms the importance of considering multiple causal effects across multiple life stages when examining the survival rates of seabirds.

## Supporting Information

Table S1 Number of macaroni penguins PIT tagged and recaptured by season with annual population size and fecundity. **Table S2.** Temporal resolution of environmental covariates. **Table S3.** Model selection for the global model structure of age class and maximum age of transition. **Table S4.** Model selection, ANODEV and LRT tests for individual trait, top-down and bottom-up covariates. Recapture and transition probabilities specified as Model 1 (Table1).

## References

[b1] Ainley DG, Ballard G, Karl BJ, Dugger KM (2005). Leopard seal predation rates at penguin colonies of different size. Antarctic Science.

[b2] Andersson M (1976). Predation and kleptoparasitism by skuas in a Shetland seabird colony. Ibis.

[b3] Atkinson A, Siegel V, Pakhomov E, Rothery P (2004). Long-term decline in krill stock and increase in salps within the Southern Ocean. Nature.

[b4] Barbraud C, Weimerskirch H (2001). Emperor penguins and climate change. Nature.

[b5] Barlow KE, Croxall JP (2002). Seasonal and interannual variation in foraging range and habitat of macaroni penguins *Eudyptes chrysolophus* at South Georgia. Marine Ecology-Progress Series.

[b6] Baum JK, Worm B (2009). Cascading top-down effects of changing oceanic predator abundances. Journal of Animal Ecology.

[b7] Bonner W, Hunter S (1982). Predatory interactions between Antarctic fur seals, macaroni penguins and giant petrels. British Antarctic Survey Bulletin.

[b8] Borboroglu PG, Boersma PD (2013). Penguins: Natural History and Conservation.

[b9] Boulinier T, Danchin E, Monnat JY, Doutrelant C, Cadiou B (1996). Timing of prospecting and the value of information in a colonial breeding bird. Journal of Avian Biology.

[b10] Brierley AS, Demer DA, Watkins JL, Hewitt RP (1999). Concordance of interannual fluctuations in acoustically estimated densities of Antarctic krill around South Georgia and Elephant Island: biological evidence of same-year teleconnections across the Scotia Sea. Marine Biology.

[b11] Burnham KP, Anderson DR (2002). Model Selection and Multimodel Inference.

[b12] Cooch E, White G (2011). Program MARK: a Gentle Introduction.

[b13] Croxall JP, Trathan PN, Murphy EJ (2002). Environmental Change and Antarctic Seabird Populations. Science.

[b14] de Brooke ML (2004). The food consumption of the world's seabirds. Proceedings of the Royal Society B Suppl.

[b15] Dehnhard N, Poisbleau M, Demongin L, Ludynia K, Lecoq M, Masello JF (2013). Survival of rockhopper penguins in times of global climate change. Aquatic Conservation: Marine and Freshwater Ecosystems.

[b16] Forcada J, Trathan PN (2009). Penguin responses to climate change in the Southern Ocean. Global Change Biology.

[b17] Frederiksen M, Daunt F, Harris MP, Wanless S (2008). The demographic impact of extreme events: stochastic weather drives survival and population dynamics in a long-lived seabird. Journal of Animal Ecology.

[b18] González-Solís J, Croxall JP, Wood AG (2000). Foraging partitioning between giant petrels Macronectes spp. and its relationship with breeding population changes at Bird Island, South Georgia. Marine Ecology-Progress Series.

[b19] Green C, Trathan P, Preston M (2006). A new automated logging gateway to study the demographics of macaroni penguins (*Eudyptes chrysolophus*) at Bird Island, South Georgia: testing the reliability of the system using radio telemetry. Polar Biology.

[b20] Grosbois V, Gimenez O, Gaillard JM, Pradel R, Barbraud C, Clobert J (2008). Assessing the impact of climate variation on survival in vertebrate populations. Biological Reviews.

[b21] Guinard E, Weimerskirch H, Jouventin P (1998). Population changes and demography of the northern Rockhopper Penguin on Amsterdam and Saint Paul Islands. Colonial Waterbirds.

[b22] Hofmann EE, Hüsrevoğlu YS (2003). A circumpolar modeling study of habitat control of Antarctic krill (*Euphausia superba*) reproductive success. Deep Sea Research Part II: Topical Studies in Oceanography.

[b23] Hofmann EE, Klinck JM, Locarnini RA, Fach B, Murphy E (1998). Krill transport in the Scotia Sea and environs. Antarctic Science.

[b24] Hunter S (1983). The food and feeding ecology of the giant petrels Macronectes halli and *M. giganteus* at South Georgia. Journal of Zoology.

[b25] Hunter S, Brooke MDL (1992). The Diet of Giant Petrels Macronectes spp. at Marion Island, Southern Indian Ocean. Colonial Waterbirds.

[b26] Jenouvrier S (2013). Impacts of climate change on avian populations. Global Change Biology.

[b27] Jenouvrier S, Barbraud C, Weimerskirch H (2005). Long-term contrasted responses to climate of two Antarctic seabird species. Ecology.

[b28] Jenouvrier S, Holland M, Stroeve J, Barbraud C, Weimerskirch H, Serreze M (2012). Effects of climate change on an emperor penguin population: analysis of coupled demographic and climate models. Global Change Biology.

[b29] Lalas C, Ratz H, Mcewan K, Mcconkey SD (2007). Predation by New Zealand sea lions (*Phocarctos hookeri*) as a threat to the viability of yellow-eyed penguins (*Megadyptes antipodes*) at Otago Peninsula, New Zealand. Biological Conservation.

[b30] Le Bohec C, Gauthier-Clerc M, Gendner JP, Chatelain N, Le Maho Y (2003). Nocturnal predation of king penguins by giant petrels on the Crozet Islands. Polar Biology.

[b31] Le Bohec C, Gauthier-Clerc M, Gremillet D, Pradel R, Bechet A, Gendner JP (2007). Population dynamics in a long-lived seabird: I. Impact of breeding activity on survival and breeding probability in unbanded king penguins. Journal of Animal Ecology.

[b32] Le Bohec C, Durant JM, Gauthier-Clerc M, Stenseth NC, Park Y-H, Pradel R (2008). King penguin population threatened by Southern Ocean warming. Proceedings of the National Academy of Sciences.

[b33] Lebreton J-D, Pradel R (2002). Multistate recapture models: modelling incomplete individual histories. Journal of Applied Statistics.

[b34] Lebreton J-D, Burnham KP, Clobert J, Anderson DR (1992). Modeling survival and testing biological hypotheses using marked animals: a unified approach with case studies. Ecological Monographs.

[b35] Loeb V, Siegel V, Holm-Hansen O, Hewitt R, Fraser W, Trivelpiece W (1997). Effects of sea-ice extent and krill or salp dominance on the Antarctic food web. Nature.

[b36] Mcclung MR, Seddon PJ, Massaro M, Setiawan AN (2004). Nature-based tourism impacts on yellow-eyed penguins *Megadyptes antipodes*: does unregulated visitor access affect fledging weight and juvenile survival?. Biological Conservation.

[b37] Meredith MP, Brandon MA, Murphy EJ, Trathan PN, Thorpe SE, Bone DG (2005). Variability in hydrographic conditions to the east and northwest of South Georgia, 1996–2001. Journal of Marine Systems.

[b38] Meredith MP, Murphy EJ, Hawker EJ, King JC, Wallace MI (2008). On the interannual variability of ocean temperatures around South Georgia, Southern Ocean: forcing by El Niño/Southern Oscillation and the Southern Annular Mode. Deep Sea Research Part II: Topical Studies in Oceanography.

[b39] Murphy EJ, Watkins JL, Reid K, Trathan PN, Everson I, Croxall JP (1998). Interannual variability of the South Georgia marine ecosystem: biological and physical sources of variation in the abundance of krill. Fisheries Oceanography.

[b40] Murphy EJ, Trathan PN, Watkins JL, Reid K, Meredith MP, Forcada J (2007). Climatically driven fluctuations in Southern Ocean ecosystems. Proceedings of the Royal Society B: Biological Sciences.

[b41] Nur N, Sydeman WJ (1999). Demographic processes and population dynamic models of seabirds. Current Ornithology.

[b42] Olsson O (1997). Effects of food availability on fledging condition and post-fledging survival in king penguin chicks. Polar Biology.

[b43] Pitman R, Durban J (2010). Killer whale predation on penguins in Antarctica. Polar Biology.

[b44] Quetin LB, Ross MM (2003). Episodic recruitment in Antarctic krill *Euphausia superba* in the Palmer LTER study region. Marine Ecology-Progress Series.

[b45] Ratcliffe N, Crofts S, Brown R, Baylis AMM, Adlard S, Horswill C (2014). Love thy neighbour or opposites attract? Patterns of spatial segregation and association among crested penguin populations during winter. Journal of Biogeography.

[b46] Reid K, Croxall JP (2001). Environmental response of upper trophic-level predators reveals a system change in an Antarctic marine ecosystem. Proceedings of the Royal Society B-Biological Sciences.

[b47] Reid K, Trathan P, Croxall J, Hill H (1996). Krill caught by predators and nets: differences between species and techniques. Marine Ecology Progress Series.

[b48] Reid T, Hindell M, Lavers JL, Wilcox C (2013). Re-examining mortality sources and population trends in a declining seabird: using Bayesian methods to incorporate existing information and new data. PLoS ONE.

[b49] Reisinger RR, De Bruyn PJN, Bester MN (2011). Predatory impact of killer whales on pinniped and penguin populations at the Subantarctic Prince Edward Islands: fact and fiction. Journal of Zoology.

[b50] Reynolds RW, Rayner NA, Smith TM, Stokes DC, Wang W (2002). an improved in situ and satellite SST analysis for climate. Journal of Climate.

[b51] Ryan PG, Sommer E, Breytenbach E (2008). Giant petrels *Macronectes* hunting Northern rockhopper penguins *Eudyptes moseleyi* at sea. Ardea.

[b52] Saraux C, Le Bohec C, Durant JM, Viblanc VA, Gauthier-Clerc M, Beaune D (2011a). Reliability of flipper-banded penguins as indicators of climate change. Nature.

[b53] Saraux C, Viblanc VA, Hanuise N, Le Maho Y, Le Bohec C (2011b). Effects of individual pre-fledging traits and environmental conditions on return patterns in juvenile king penguins. PLoS ONE.

[b54] Schielzeth H (2010). Simple means to improve the interpretability of regression coefficients. Methods in Ecology and Evolution.

[b55] Schwarz LK, Goebel ME, Costa DP, Kilpatrick AM (2013). Top-down and bottom-up influences on demographic rates of Antarctic fur seals *Arctocephalus gazella*. Journal of Animal Ecology.

[b56] Sengupta A, England MH (2006). Coupled Ocean–Atmosphere–Ice Response to Variations in the Southern Annular Mode. Journal of Climate.

[b57] Sidhu LA, Dann P, Chambers L, Catchpole EA (2012). Seasonal ocean temperature and the survival of first-year little penguins *Eudyptula minor* in south-eastern Australia. Marine Ecology-Progress Series.

[b58] Siegel V (2005). Distribution and population dynamics of *Euphausia superba*: summary of recent findings. Polar Biology.

[b59] Skalski JR, Hoffmann A, North PM, Smith SG, Lebreton J-D, North PM (1993). Testing the significance of individual- and cohort-level covariates in animal survival studies. Marked Individuals in the Study of Bird Population.

[b60] Smith CA, Sardeshmukh P (2000). The effect of ENSO on the intraseasonal variance of surface temperature in winter. International Journal of Climatology.

[b61] Spendelow JA, Nichols JD, Hines JE, Lebreton J-D, Pradel R (2002). Modelling postfledging survival and age-specific breeding probabilities in species with delayed maturity: a case study of Roseate Terns at Falkner Island, Connecticut. Journal of Applied Statistics.

[b62] Staniland I, Robinson SL, Silk JRD, Warren N, Trathan PN (2012). Winter distribution and haul-out behaviour of female Antarctic fur seals from South Georgia. Marine Biology.

[b63] Trathan PN, Ratcliffe N, Masden EA (2012). Ecological drivers of change at South Georgia: the krill surplus, or climate variability. Ecography.

[b64] Trathan PN, Everson I, Murphy EJ, Parkes GB (1998). Analysis of haul data from the South Georgia krill fishery. CCAMLR Science.

[b65] Trathan PN, Brierley AS, Brandon MA, Bone DG, Goss C, Grant SA (2003). Oceanographic variability and changes in Antarctic krill (*Euphausia superba*) abundance at South Georgia. Fisheries Oceanography.

[b66] Trathan PN, Green C, Tanton J, Peat H, Poncet J, Morton A (2006). Foraging dynamics of macaroni penguins *Eudyptes chrysolophus* at South Georgia during brood-guard. Marine Ecology-Progress Series.

[b67] Turner J (2004). Review: the El Niño/Southern Oscillation and Antarctica. International Journal of Climatology.

[b68] Waluda CM, Hill SL, Peat HJ, Trathan PN (2012). Diet variability and reproductive performance of macaroni penguins *Eudyptes chrysolophus* at Bird Island, South Georgia. Marine Ecology-Progress Series.

[b69] Williams TD (1992). Reproductive endocrinology of macaroni (*Eudyptes chrysolophus*) and gentoo (*Pygoscelis papua*) penguins: I. Seasonal changes in plasma levels of gonadal steroids and LH in breeding adults. General and Comparative Endocrinology.

[b70] Williams TD (1995). The Penguins: Spheniscidae.

