# Supplementary material for: Survival in macaroni penguins and the relative importance of different drivers: individual traits, predation pressure and environmental variability
Source: J Anim Ecol. 2014 May 21;83(5):1057–67. doi: 10.1111/1365-2656.12229 (PMC4284017; doi:10.1111/1365-2656.12229)
Supplement: Table S1 — Number of macaroni penguins PIT tagged and recaptured by season with annual population size and fecundity. Table S2. Temporal resolution of environmental covariates. Table S3. Model selection for the global model structure of age class and maximum age of transition. Table S4. Model selection, ANODEV and LRT tests for individual trait, top-down and bottom-up covariates. Recapture and transition probabilities specified as Model 1 (Table1). [file jane0083-1057-SD1.doc]

S1. Number of macaroni penguins PIT tagged and recaptured by season with annual population size and fecundity.

| Season | Total no. birds tagged >1 yr old | Total no. fledglings tagged | Total no. individuals recaptured by gateway | Total d gateway in operation | Total no. individuals recaptured manually | Colony size | |
| --- | --- | --- | --- | --- | --- | --- | --- |
|  |  |  |  |  |  | No. breeding pairs | No. chicks fledged |
| 2003 | 114 | 76 | - | - | - | 536 | 315 |
| 2004 | 362 | 106 | 113 | 148 | 0 | 463 | 264 |
| 2005 | 151 | 118 | 431 | 154 | 0 | 537 | 272 |
| 2006 | 16 | 102 | 535 | 158 | 0 | 365 | 181 |
| 2007 | 52 | 100 | 0 | 0 | 79 | 527 | 243 |
| 2008 | 0 | 100 | 554 | 146 | 158 | 521 | 367 |
| 2009 | 90 | 120 | 470 | 14 | 175 | 511 | 372 |
| 2010 | 27 | 100 | 604 | 110 | 0 | 431 | 212 |
| 2011 | 90 | 96 | 599 | 120 | 1 | 409 | 263 |
| 2012 | 64 | 152 | 630 | 150 | 422 | 493 | 237 |
| Total | 966 | 1070 | 3936 | 1000 | 835 | - | - |

S2. Temporal resolution of environmental covariates.

| No. | Covariate | Study period | Time lag (years) | Parameter |
| --- | --- | --- | --- | --- |
| 1 | Local sea surface temperature anomalies (LSST) | Annual (October *t-1* to September *t*)  Annual (October *t-2* to September *t-1*) | 0  1 | LSST_0_  LSST_-1_ |
| 2 | El Niño/Southern Oscillation Index (ENSO) | Summer (October *t-3* to March *t-2*)  Summer (October *t-4* to March *t-3*) | 2  3 | ENSO_-2_  ENSO_-3_ |
| 3 | Southern Annular Mode index (SAM) | Summer (October *t-1* to March *t*)  Summer (October *t-2* to March *t-1*) | 0  1 | SAM_0_  SAM_-1_ |

Table S3. Model selection for the global model structure of age class and maximum age of transition

| Model | AIC_c_ |  | ΔAIC_c_ | k | Model  deviance |
| --- | --- | --- | --- | --- | --- |
| *a3 a*t / a*t p_0 / t_ Ψ_3 / 0_* | 1843.69 |  | 27.32 | 40 | 1785.35 |
| *a3 a*t / a*t p_0 / t_ Ψ_4 / 0_* | 1847.66 |  | 31.29 | 41 | 1820.37 |
| *a1 t p_0 / t_ Ψ_4 / 0_* | 1849.85 |  | 33.48 | 24 | 1911.70 |
| *a1 t p_0 / t_ Ψ_3 / 0_* | 2018.63 |  | 202.26 | 23 | 1982.50 |

Notes; Model fit is assessed using the lowest AIC_C_, with the difference between the best candidate model (Table 1) and other models specified (ΔAIC_c_ ). See Table 1 for notation details.

S4. Model selection, ANODEV and LRT tests for individual trait, top-down and bottom-up covariates. Recapture and transition probabilities specified as Model 2 (Table 2).

| Step | Model | k | ANODEV tests for cohort-level covariates | | | |  |
| --- | --- | --- | --- | --- | --- | --- | --- |
|  |  |  | *df* | *F* | *P* | R^2^ |  |
| 3 | Mass + ENSO_-3_ / ENSO_-3_ | 12 | 1 | 0.70 | 0.41 | 0.04 | |
| 3 | Mass + μ Mass / . | 12 | 1 | 0.90 | 0.35 | 0.06 | |
| 3 | Mass + LSST_0_ / LSST_0_ | 12 | 1 | 1.15 | 0.30 | 0.07 | |
| 3 | Mass + SAM_-1_ / SAM_-1_ | 12 | 1 | 1.54 | 0.23 | 0.09 | |
| 3 | Mass + SAM_0_ / SAM_0_ | 12 | 1 | 2.24 | 0.15 | 0.13 | |
| 3 | Mass + ENSO_-2_ / ENSO_-2_ | 12 | 1 | 3.49 | 0.08 | 0.19 | |
| 3 | Mass + LSST_-1_ / LSST_-1_ | 12 | 1 | 5.89 | 0.03 | 0.28 | |
| 4 | Mass + ENSO_-3_ / ENSO_-3_ ** | 13 | 1 | 0.10 | 0.75 | 0.01 | |
| 4 | Mass + LSST_-1_ / LSST_-1_ ** | 13 | 1 | 0.72 | 0.41 | 0.05 | |
| 4 | Mass + SAM_-1_ / SAM_-1_ ** | 13 | 1 | 1.55 | 0.23 | 0.10 | |
| 4 | Mass + SAM_0_ / SAM_0_ ** | 13 | 1 | 1.79 | 0.20 | 0.11 | |
| 4 | Mass + LSST_0_ / LSST_0_ ** | 13 | 1 | 2.01 | 0.18 | 0.13 | |
| 4 | Mass + ENSO_-2_ / ENSO_-2_ ** | 13 | 1 | 4.12 | 0.06 | 0.23 | |
| 5 | Mass + Pred. + LSST_0_ / Pred. + LSST_0_ | 14 | 1 | 0.31 | 0.74 | 0.00 | |
| 5 | Mass + Pred. + ENSO_-2_ / Pred. + ENSO_-2_ | 14 | 1 | 1.65 | 0.23 | 0.03 | |
| 5 | Mass + Pred. + SAM_-1_ / Pred. + SAM_-1_ | 14 | 1 | 2.88 | 0.11 | 0.18 | |
| 5 | Mass + Pred. + ENSO_-3_ / Pred. + ENSO_-3_ | 14 | 1 | 3.19 | 0.10 | 0.20 | |
| 5 | Mass + Pred. + SAM_0_ / Pred. + SAM_0_ | 14 | 1 | 7.40 | 0.02 | 0.36 | |
| 6 | Mass + Pred. + SAM_0_ / Pred. + SAM_0_ ** | 15 | 1 | 0.26 | 0.61 | 0.02 | |
| 6 | Mass + Pred. + SAM_-1_ / Pred. + SAM_-1_ ** | 15 | 1 | 0.47 | 0.50 | 0.04 | |
| 6 | Mass + Pred. + LSST_0_ / Pred. + LSST_0_ ** | 15 | 1 | 0.61 | 0.45 | 0.05 | |
| 6 | Mass + Pred. + LSST_-1_ / Pred. + LSST_-1_ ** | 15 | 1 | 0.73 | 0.41 | 0.06 | |
| 6 | Mass + Pred. + ENSO_-3_ / Pred. + ENSO_-3_ ** | 15 | 1 | 1.37 | 0.27 | 0.10 | |
| 6 | Mass + Pred. + ENSO_-2_ / Pred. + ENSO_-2_ ** | 15 | 1 | 2.77 | 0.12 | 0.19 | |
| 6 | Mass + Pred. + LSST_-1_ + ENSO_-3_ /  Pred. + LSST_-1_ + ENSO_-3_ | 15 | 1 | 0.37 | 0.55 | 0.03 | |
| 6 | Mass + Pred. + LSST_-1_ + SAM_-1_ /  Pred. + LSST_-1_ + SAM_-1_ | 15 | 1 | 0.40 | 0.54 | 0.03 | |
| 6 | Mass + Pred. + LSST_-1_ + ENSO_-2_ /  Pred. + LSST_-1_ + ENSO_-2_ | 15 | 1 | 0.60 | 0.46 | 0.05 | |
| 6 | Mass + Pred. + LSST_-1_ + μ Mass /  Pred. + LSST_-1_ + μ Mass | 15 | 1 | 0.61 | 0.45 | 0.05 | |

Notes; Predation pressure (Pred.) included as an interactive effect with age class, and environmental covariates considered as additive with age class unless specified. ** step-up from an additive effect to an interactive effect with age class for the covariate being considered. Bottom-up covariates considered at specified lags. (μ Mass) annual mean of fledging mass.
